# Intrinsically Conductive and Cu‐Functionalized Polymer‐Composite Membranes as Gas Diffusion Electrodes for CO_2_ Electroreduction

**DOI:** 10.1002/cssc.202401228

**Published:** 2024-10-24

**Authors:** Ignacio Sanjuán, Vaibhav Kumbhar, Oleg Prymak, Mathias Ulbricht, Corina Andronescu, Lukas Fischer

**Affiliations:** ^1^ Technische Chemie III Universität Duisburg-Essen Carl-Benz-Straße 199 47057 Duisburg Germany; ^2^ Lehrstuhl für Technische Chemie II Universität Duisburg-Essen Universitätsstr. 7 45141 Essen Germany; ^3^ Anorganische Chemie Universität Duisburg-Essen Universitätsstr. 5 45141 Essen Germany; ^4^ Center for Nanointegration Duisburg–Essen (CENIDE) Universität Duisburg-Essen Carl-Benz-Str. 199 47057 Duisburg Germany; ^5^ Center for Water and Environmental Research (ZWU) Universität Duisburg-Essen Universitätsstr. 2 45141 Essen Germany

**Keywords:** membranes, gas diffusion electrodes, carbon dioxide fixation, microenvironment, energy conversion

## Abstract

We introduced a new class of gas diffusion electrodes (GDEs) with adjustable pore morphology. We fabricated intrinsically conductive polymer‐composite membranes containing carbon filler, enabling a pore structure variation through film casting *cum* phase separation protocols. We further selectively functionalized specific pore regions of the membranes with Cu by a NaBH_4_‐facilitated coating strategy. The as‐obtained GDEs can facilitate the electrochemical CO_2_ reduction reaction (CO_2_RR) at Cu active sites that are presented inside a defined and electrically conductive pore system. When employing them as free‐standing cathodes in a CO_2_ flow electrolyzer, we achieved >70 % Faradaic efficiencies for CO_2_RR products at up to 200 mA/cm^2^. We further demonstrated that deposition of a dense Cu layer on top of the membrane leads to obstruction of the underlying pore openings, inhibiting an excessive wetting of the pore pathways that transport gaseous CO_2_. However, the presentation of Cu inside the pore system of our novel membrane electrodes increased the C_2_H_4_/CO selectivity by a factor of up to 3 compared to Cu presented in the dense layer on top of the membrane. Additionally, we found that gaseous CO_2_ could still access Cu in macropores after wetting with electrolyte, while CO_2_RR was completely suppressed in wetted nm‐scale pores.

## Graphical abstract

We established intrinsically conductive and Cu‐functionalized polymer‐composite membranes as gas diffusion electrodes with adjustable morphology. When used as cathodes in a CO_2_ flow electrolyzer, these membrane electrodes enable CO_2_ electroreduction in a tailored pore environment. Overall, we demonstrated that Cu presentation inside electrically conductive macropores can increase the C_2_H_4_/CO selectivity.

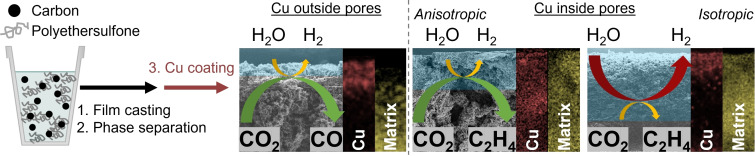

In light of increasing energy demands and human‐made climate change, there is an urgent need for technologies that use electricity from renewable sources to transform abundant small molecules into essential chemicals and fuels.[^1^, ^2^] In this context, different electrolyzer technologies have emerged, for example for water, CO_2_ and N_2_ electrolysis.[^3^, ^4^, ^5^] However, the latter two still face significant challenges. Among the main limitations are deficits in the design of effective gas diffusion electrodes (GDEs) as porous contactors between the catalyst, the electrolyte and the gaseous feed.[^6^, ^7^, ^8^] For use in the electrochemical CO_2_ reduction reaction (CO_2_RR), most GDEs are prepared by coating a thin metal layer onto the outer surface of a gas diffusion layer (GDL), which means that the catalyst sites are densely clustered and in direct contact to the electrolyte during CO_2_RR.[^9^, ^10^] A key factor that determines the overall performance under these conditions is the catalyst microenvironment, specifically the local gas‐liquid transport.[^11^, ^12^, ^13^, ^14^, ^15^] This involves the distribution of electrolyte to catalyst sites in deeper regions of the coated metal layer while preventing the transport pathways for gaseous CO_2_ from being blocked by excessive wetting with liquid. As a result of this state‐of‐the‐art GDE design, investigations have primarily focused on tuning the transport properties by enhancing the hydrophobicity, for example through the coating of a hydrophobic polymer onto the metal catalyst.[^16^, ^17^, ^18^, ^19^] Another potential approach to alter the gas‐liquid transport around catalyst sites could be to place the metal inside the pore network of a GDL. This would allow to tune the mass transport by exploiting the influence of the pore size distribution on the capillary pressure at the gas‐liquid interface.^[20]^ However, this approach is currently limited by the availability of electrodes that can facilitate CO_2_RR within a pore system with an adjustable structure.^[21]^ Electrically conductive GDLs, such as carbon paper or metal felts, can be fully functionalized with catalysts, thereby enabling CO_2_RR within the GDL itself.^[22]^ However, these GDLs exhibit a wide pore size distribution, which cannot be fine‐tuned. Alternatively, porous polymer membranes that are prepared via solidification of a matrix polymer from solution through phase separation could be promising GDLs with an adjustable pore structure, but their electrical non‐conductivity prevents the facilitation of CO_2_RR within their pore systems.[^23^, ^24^, ^25^, ^26^]

To impart electrical conductivity, a conductive filler would need to be incorporated into the polymer matrix. As of yet, there are only few reports in literature of the successful incorporation of enough filler to reach percolation throughout the entire polymer membrane while maintaining the mechanical integrity and a defined pore structure.[^27^, ^28^] Additionally, the few examples that do exist are relatively specialized use cases, and no attempts have been made to develop an electrode platform based on this approach.

In this work, we present the fabrication of intrinsically conductive polymer‐composite membranes with anisotropic and isotropic pore structures, providing a valuable toolkit for the preparation of thin‐film electrodes with potential use in a variety of electrochemical applications.

We further developed a coating strategy based on differences in gas‐liquid transport inside the pore system to functionalize these membranes selectively with Cu in certain regions. As proof‐of‐concept, we employed our GDEs in a CO_2_RR flow electrolyzer. We could demonstrate that presentation of copper inside the electrically conductive pore network can increase the C_2_H_4_/CO product selectivity by a factor of up to 3 compared to copper presented outside the pore system. Additionally, we could show that macrovoids in the GDEs promote the transport of gaseous CO_2_ to Cu sites after being wetted with electrolyte, enabling CO_2_RR inside the conductive pores. In contrast, pores with nm‐scale diameter inhibited access of CO_2_ to Cu following pore wetting, thereby completely suppressing CO_2_RR activity. These novel insights may serve as a general roadmap for the design of GDEs with improved CO_2_RR performance.

The fabrication of conductive polymer‐composite membranes via film casting *cum* phase separation is presented in Figure 1. This method was developed through an in‐depth investigation of carbon fillers, dispersing additives, and homogenization methods (see SI, Section S2).

**Figure 1:**
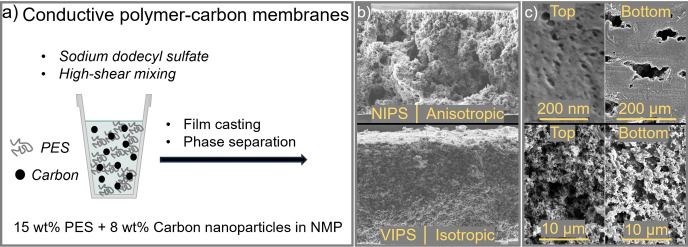
a) Fabrication of porous and intrinsically conductive polymer‐carbon membranes via NIPS (anisotropic) or VIPS (isotropic) (see SI, Section S2–3). b) SEM images of the cross sections. c) SEM images of the top and bottom surfaces.

The final process is based on casting dispersions consisting of 15 wt% polyethersulfone (PES) as non‐fluorinated matrix polymer, 8 wt% carbon nanoparticles (Vulcan XC72) as conductive filler and 1 wt% sodium dodecyl sulfate as dispersing agent, with N‐methyl‐2‐pyrrolidone (NMP) as solvent (Figure 1a).[^29^, ^30^, ^31^] Utilizing established film casting *cum* phase separation protocols enabled us to adjust the pore morphology (Figure 1b). Conducting the phase separation of cast films in either liquid water (liquid non‐solvent induced phase separation, NIPS) or water vapor (non‐solvent vapor induced phase separation, VIPS) yielded electrically conductive (~0.20 S/cm) anisotropic or isotropic porous membranes (see SI, Section S2). The anisotropic structure exhibits a gradient from small pore openings on the top side (≤50 nm), which is the side that had contact to the water precipitation bath during fabrication, to large macrovoids on the bottom side (≤100 μm) (Figure 1b–c). In contrast, the isotropic membrane has a sponge‐like structure and pore openings with similar diameter on both sides (~5 μm). Both these morphologies are as‐expected for membranes obtained from NIPS or VIPS after casting a film of the same polymer solution.[^32^, ^33^]

The base membranes were subjected to a newly developed iterative copper coating (Figure 2 and Section S3 in the SI). For *An_CuAq* and *Iso_CuAq*, each coating step consisted of wetting with aqueous Cu^2+^ solution, directly followed by placement in an aqueous NaBH_4_ reduction bath. During this process, an exponential increase in Cu loading occurred after each coating step (Figure 2, top). This could be attributed to enhanced adsorption of Cu^2+^ ions on the already deposited Cu(0). Initially, the conductivity of the membranes exhibited minimal changes. However, a sudden conductivity increase was observed after the Cu loading surpassed 1 mg/cm^2^, marking the percolation point of copper on the membrane surface. In contrast, a bare PES membrane without carbon filler achieved a comparable copper uptake during coating but showed no electrical conductivity at high Cu loading (see SI, Section S4). This demonstrates the critical role of the conductive carbon network in bridging gaps in the deposited copper. After completion of the coating process, a copper film (~20 μm) formed on the top surface of the anisotropic *An_CuAq* (Figure 2, left). In the reduction bath, the open pore structure at the bottom (cf. Figure 1c) likely promoted entry of NaBH_4_ into the wetted (aqueous Cu^2+^) membrane, resulting in reduction of Cu^2+^ to Cu(0) in the lower pore region. Additionally, H_2_ gas generation from NaBH_4_ hydrolysis may lead to a pressure increase in the pore system, pushing the remaining Cu^2+^ solution out of the pores on the top side, followed by Cu(0) film deposition in contact to the surrounding reduction solution. In contrast, for *Iso_CuAq*, only the upper and lower parts of the pore system were copper coated (Figure 2, middle). The isotropic morphology facilitates the diffusion of NaBH_4_ from both sides into the wetted membrane, and H_2_ gas bubble formation in the pores may have blocked NaBH_4_ diffusion into deeper regions, resulting in a limited coating depth. In an alternative coating process, *An_CuEt* was first dried after wetting with Cu^2+^ in ethanol and was then placed in NaBH_4_ in ethanol. This led to the deposition of a constant amount of copper with each coating step and a homogeneous Cu distribution through the cross section (Figure 2, right). Additionally, copper percolation was already reached at a Cu loading of ~0.5 mg/cm^2^.

**Figure 2:**
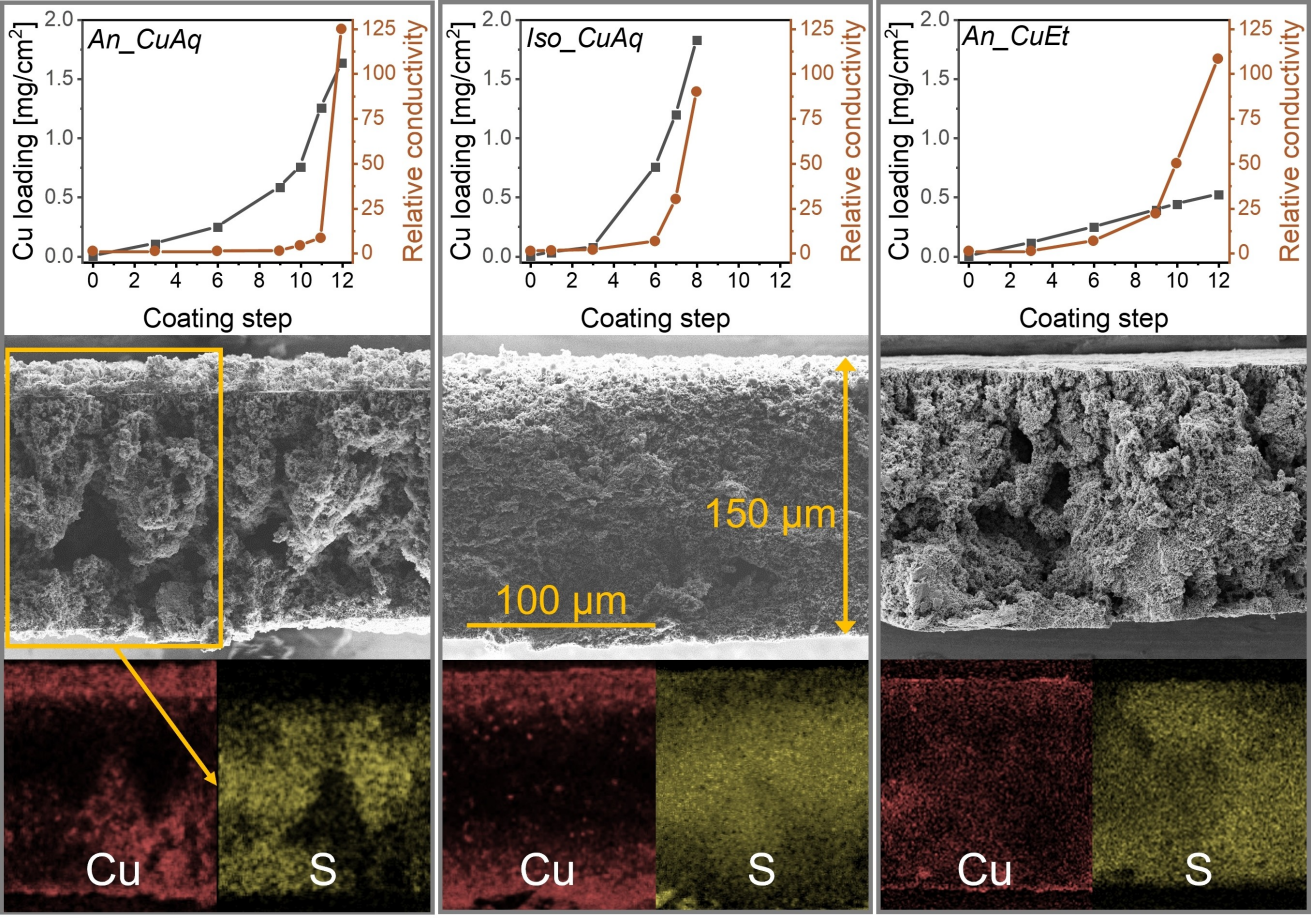
Cu loading and relative conductivity increase during the iterative Cu coating procedure (top) as well as SEM images (middle) and EDX mapping (bottom) of the final GDEs (after the last coating step, see Section S3 in the SI for details). *An_CuAq* (anisotropic) and *Iso_CuAq* (isotropic) were obtained from an iterative coating in aqueous CuCl_2_ and aqueous NaBH_4_. *An_CuEt* (anisotropic) was obtained from an iterative coating in Cu(OAc)_2_ in ethanol and NaBH_4_ in ethanol. The exemplary scale bar shown in the middle SEM image corresponds to all SEM images and the exemplary area marking in the left SEM image corresponds to all EDX mappings.

All final GDEs (cf. Figure 2, obtained after last coating step) exhibit an identical XRD pattern with copper peaks at 43.5°, 50.5°, and 74°, which can be assigned to the (111), (200), and (220) crystal lattice planes of Cu(0) (Figure 3a).^[34]^ The peak intensities further correlate with the copper loading (cf. Figure 2), and the Rietveld refinement yielded similar lattice parameters and crystallite sizes (see SI, Section S5). The normalized Cu 2p XPS spectra also demonstrate an identical chemical state of copper in all GDEs (Figure 3b), and the absence of Cu^2+^ satellite peaks at 940 eV confirms the deposition of Cu(0).^[35]^ For the base membrane, the two broad XRD peaks between 15–30° can be assigned to the PES matrix and the incorporated carbon nanoparticles, respectively (Figure 3a). Additionally, the deconvoluted C 1s XPS signal of the base membrane (Figure 3c) depicts the presence of C=O and COOH groups on the top side (~5 nm XPS analysis depth). These groups are exclusive to the carbon nanoparticles, indicating an embedment of conductive carbon close to the surface of the PES matrix.

**Figure 3:**
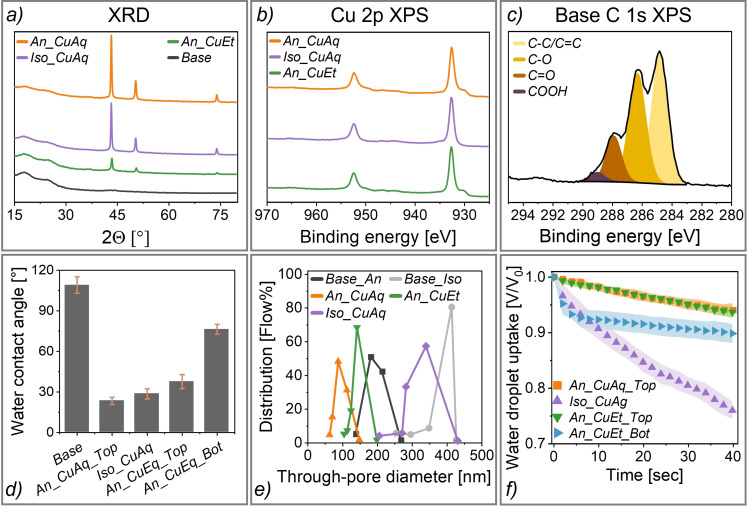
a) XRD patterns. b) Normalized XPS Cu 2p spectra (top side). c) Fitted XPS C 1s spectrum of the base membrane (top side). d) Surface water contact angles (Base: average of both sides of both base membranes). e) Through‐pore diameter distribution obtained from capillary flow porometry. f) Surface water droplet uptake. Average and standard deviation obtained from three different samples. For experimental details, see Section S5–7 in the SI.

The water contact angle data illustrates a hydrophilization after Cu coating, lowering the average contact angle from 109° for the base membranes to 23–38° for the top side of the functionalized GDEs (Figure 3d). Notably, the contact angle of the bottom side of *An_CuEt* is significantly higher (76°), demonstrating a lower Cu deposition on the outer bottom surface. We further determined the through‐pore diameter distribution of the GDEs before and after the Cu coating via pore dewetting capillary flow porometry (Figure 3e). For this technique, the pore system was first fully wetted with a perfluorinated liquid (Galwick®), followed by displacement of the liquid at step‐wise increasing gas transmembrane pressure. From the pressure at which liquid is expelled from the pore system, the diameter of the through‐pores can be calculated via Washburn's equation (see SI, Section S6). The increase of gas flow at each pressure is related to the fraction of pores with that diameter. However, without further corrections the resulting pore size distribution does not exactly correspond to the number distribution; it is biased by the influence of the pore size on the specific resistance to gas flow.^[36]^ It is also important to note that this calculation assumes an ideal cylindrical pore geometry, which leads to an overestimation of the diameter when pores deviate from this shape, in particular when the length is similar to the diameter.^[37]^ This may also explain why the mean through‐pore diameter of around 200 nm that we observed for the anisotropic base membrane is significantly larger than the pore openings observed in the surface SEM image (cf. Figure 1). Moreover, the diameter obtained from this technique only corresponds to the narrowest section in each through‐pore, because this section is limiting for the liquid displacement. For the isotropic base membrane, the mean through‐pore diameter is approximately 400 nm (Figure 3e), which contrasts with the μm‐sized pore openings on the outer surface (cf. Figure 1). This indicates that the narrowest section of each pore pathway is located inside the isotropic membrane.

After the Cu coating (cf. Figure 2), the mean through‐pore diameter slightly decreased from around 400 nm to 350 nm for *Iso_CuAq* (Figure 3e). In contrast, for both anisotropic GDEs, a more pronounced pore constriction occurred during Cu coating. The mean through‐pore diameter decreased from approximately 200 nm to 150 nm for *An_CuEt* and 100 nm for *An_CuAq*, respectively. We also analyzed the effective water contact angle inside the pore network of the GDEs (see SI, Section S6). For this, we compared the pressure needed for displacement of Galwick® from the pores, which is a fully wetting liquid with a contact angle of 0°, to the pressure needed for displacement of water. For *An_CuEt and Iso_CuAq*, the thereby determined contact angles decreased from 70–80° for the largest diameter through‐pores to less than 40° for the smallest ones (see SI, Table S4). This likely illustrates that the thin through‐pores are the result of a pore constriction by hydrophilic Cu that was deposited inside these GDEs, which aligns with the Cu localization observed in the EDX mapping (cf. Figure 2). In contrast, *An_CuAq* exhibits contact angles of >60° in all through‐pores. This indicates that the decrease of the pore diameter in *An_CuAq* (cf. Figure 3e) results from the obstruction of the pore openings by the thick Cu layer on the outer top surface, and that little Cu was coated inside the pores in the upper membrane region (cf. Figure 2). The surface water droplet uptake behaviour further reveals a fast and continuous uptake into the pore system of *Iso_CuAq* (Figure 3f). In contrast, the top surfaces of *An_CuAq* and *An_CuEt* exhibit a delayed wetting, which can be attributed to the smaller pore openings in the top region of these anisotropic membranes (cf. Figure 1). Surprisingly, the bottom side of the anisotropic *An_CuEt* exhibited an initial rapid water uptake, followed by a stagnant wetting. An explanation could be that small pore pathways in contact to the macrovoids act as transport bottlenecks, limiting further distribution of liquid after the initial wetting. Additionally, we observed that *An_CuAq* and both sides of *An_CuEt* exhibit a similar and neglectable surface wetting over time in contact to a water droplet, while a rapid droplet spreading occurred on the surface of *Iso_CuAq* (see SI, Section S7). This may be connected to the uneven outer surface structure of the isotropic membrane (cf. Figure 1). A higher surface roughness is known to further promote wetting with water for intrinsically hydrophilic surfaces.^[38]^

As proof‐of‐concept, we employed our GDEs as free‐standing cathodes in a flow‐cell setup for CO_2_RR with 1 M KOH as electrolyte (Figure 4 and Section S8 in the SI).

**Figure 4:**
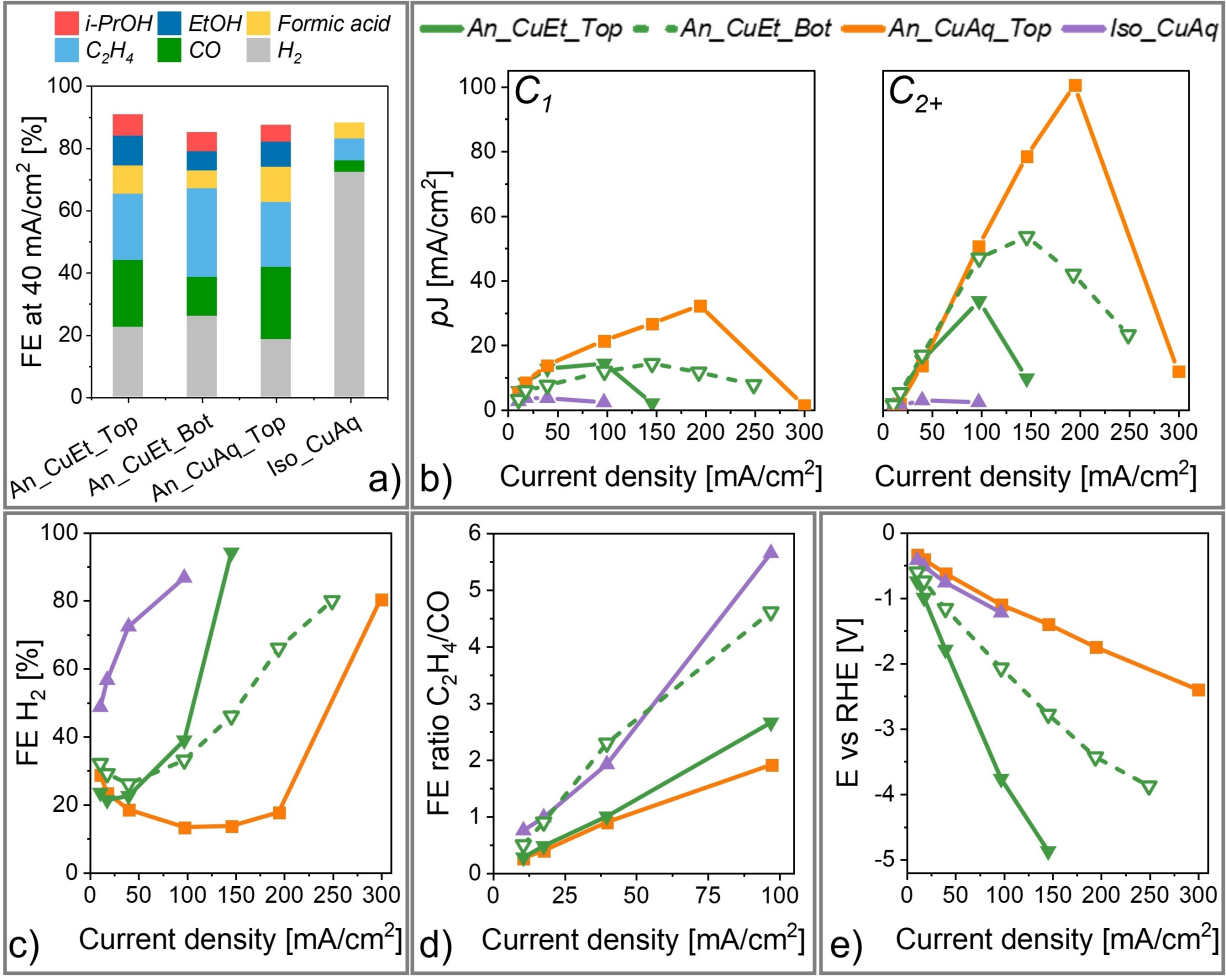
Electrochemical CO_2_RR at different current densities (galvanostatic) in a flow‐cell (1 M KOH electrolyte) using the Cu‐functionalized GDEs as free‐standing cathodes (see SI, Section S8 for details). The GDEs were oriented with either the top (*Top*) or bottom (*Bot*) side towards the electrolyte (cf. Figure 1). Current density was step‐wise increased, and reaction was monitored for 420 s for each step. Each experiment was stopped after 80 % FE for H_2_ was reached. a) Exemplary product profiles at 40 mA/cm^2^. b) Partial current densities (*p*J) for C_1_ and C_2+_ products. c) FE for H_2_. d) FE ratio of C_2_H_4_/CO e) Potential‐current curves. For assignment of data to the corresponding GDEs see legend in b).

At 40 mA/cm^2^, *An_CuEt* (tested with both sides facing electrolyte) and *An_CuAq_Top* (top side facing electrolyte) exhibited a similar CO_2_RR product profile and a low H_2_ faradaic efficiency (FE), while *Iso_CuAq* produced H_2_ with over 70 % FE (Figure 4a–c). Generally, an excessive initial pore wetting of the GDE can lead to the immersion of a large number of Cu sites in the electrolyte, promoting hydrogen evolution reaction (HER) during CO_2_RR due to an inhibited access of gaseous CO_2_. Indeed, *Iso_CuAq* exhibited the fastest water droplet uptake of all GDEs (cf. Figure 3f), aligning with the high H_2_ FE. In contrast, the anisotropic GDEs achieved higher partial current densities for C products and, consequently, a lower H_2_ production (Figure 4b).

We further observed significant differences in performance depending on the orientation for *An_CuEt*, which presents Cu in the whole pore system (cf. Figure 2). This variation reflects the impact of the anisotropic pore size gradient in this GDE (cf. Figure 1). For *An_CuEt*_*Top*, a sudden increase in H_2_ FE was observed after 100 mA/cm^2^, indicating a complete suppression of CO_2_RR activity after wetting of the upper pore region (nm pore diameter) (Figure 4c).^[39]^ However, for *An_CuEt_Bot*, oriented with the bottom side (μm pore diameter) towards the electrolyte, a slower H_2_ FE increase occurred. This aligns with the water droplet uptake of the bottom side, characterized by a fast initial uptake of part of the volume but a stagnant wetting afterwards (cf. Figure 3f). Additionally, in the bottom region, the low capillary pressure in wetted macrovoids may facilitate entry of CO_2_ gas bubbles (cf. Figure 1). This is in contrast to the top region, where the smaller pore diameters present a higher liquid capillary pressure, which gas bubbles need to overcome after pore wetting. Remarkably, this also significantly enhanced C_2+_ product formation in *An_CuEt_Bot* in comparison to *An_CuEt_Top* (Figure 4b). Moreover, *An_CuEt*_*Bot* achieved a ~3 times higher C_2_H_4_/CO ratio than *An_CuEt_Top* and *An_CuAq_Top* (Figure 4d). For *An_CuEt*_*Bot*, the liquid‐gas interface likely formed inside the pore system, indicating that this microenvironment favours a higher degree of C−C coupling during CO_2_RR. Interestingly, *Iso_CuAq*, which exhibited a fast wetting and low CO_2_RR activity, matched the high C_2_H_4_/CO ratio of *An_CuEt*_*Bot*. This strengthens the indication that Cu sites presented in a wetted pore system promote C_2_H_4_ selectivity.

Figure 4e further reveals that *An_CuEt* exhibited higher potentials during CO_2_RR than *Iso_CuAq and An_CuAq*_*Top*. This can be attributed to the lower Cu loading of *An_CuEt* (cf. Figure 2), resulting in a lower electrochemically active surface area and, therefore, higher electrical resistance (see SI, Figure S5–S8). Nevertheless, *An_CuEt_Top* and *An_CuAq_Top* depict almost identical C_2_H_4_/CO ratios (Figure 4d), which is in contrast to the up to 4 times higher potentials we observed for *An_CuEt_Top*. Usually, the electrical potential is considered to be the main driving force for the CO_2_RR product selectivity of identical catalyst sites (Figure 3).^[40]^ During CO_2_RR, the delayed water uptake of both *An_CuEt_Top* and *An_CuAq_Top* (cf. Figure 3f) likely led to the formation of the gas‐liquid interface at the contact between outer top surface and electrolyte, suggesting that the similar Cu microenvironment is responsible for the identical C_2_H_4_/CO selectivity. However, *An_CuAq_Top* achieved a significantly higher CO_2_RR performance at higher current densities, maintaining a H_2_ production below 15 % FE up to 200 mA/cm^2^. This can be related to the dense Cu layer on top of this GDE (cf. Figure 2), which obstructed the pore openings of the underlying pore system (cf. Figure 3e), and may have inhibited electrolyte entry during CO_2_RR. Here, it should be noted that *An_CuAq* presents a similar structure as conventional GDEs made via metal deposition on a non‐conductive polymer membrane, illustrating that pore blocking of the GDL can be a positive side effect of this fabrication approach.[^41^, ^42^] However, a significant drawback is the excess deposition of metal required to achieve this effect. Specifically, in our study, *An_CuAq* contains approximately three times more copper than *An_CuEt* (cf. Figure 2).

In conclusion, our results highlight the drastic impact of the pore environment on the CO_2_RR performance of otherwise identical Cu sites. Our data suggests that presentation of copper in an electrolyte wetted electrically conductive pore system can promote C−C coupling, increasing the C_2_H_4_/CO selectivity by a factor of up to 3. Moreover, we found that a large pore size is necessary to enable the transport of gaseous CO_2_ to Cu sites after electrolyte entered the pore. Additionally, we could show that an anisotropic structure inhibits liquid distribution in the pore system, which likely leads to a lower number of CO_2_ transport pathways that are blocked by excessive wetting with electrolyte. However, the best performing GDE (*p*J_C2+_ of 100 mA/cm^2^ at 200 mA/cm^2^ and −1.7 V vs. RHE) was obtained by coating a dense Cu film on the outer surface (top side with nm pore openings) of an anisotropic membrane. The dense Cu layer may inhibit the uptake of electrolyte into the pore system of the underlying gas‐distributing membrane, which could further limit the blocking of CO_2_ transport pathways through electrowetting at higher current densities.^[43]^

## Conflict of Interests

The authors declare no conflict of interest.

## Supporting information

As a service to our authors and readers, this journal provides supporting information supplied by the authors. Such materials are peer reviewed and may be re‐organized for online delivery, but are not copy‐edited or typeset. Technical support issues arising from supporting information (other than missing files) should be addressed to the authors.

Supporting Information

## Data Availability

The data that support the findings of this study are available from the corresponding author upon reasonable request.
