# Supplementary material for: Intrinsically Conductive and Cu‐Functionalized Polymer‐Composite Membranes as Gas Diffusion Electrodes for CO2 Electroreduction
Source: ChemSusChem. 2024 Oct 24;18(2):e202401228. doi: 10.1002/cssc.202401228 (PMC11739840; doi:10.1002/cssc.202401228)
Supplement: Supplementary file 1 — Supporting Information [file CSSC-18-e202401228-s001.pdf]

ChemSusChem

Supporting Information

Intrinsically Conductive and Cu-Functionalized Polymer-Composite Membranes as Gas Diffusion Electrodes for CO_2 Electroreduction

Ignacio Sanjuán, Vaibhav Kumbhar, Oleg Prymak, Mathias Ulbricht, Corina Andronescu, and Lukas Fischer*

Supporting Information

Intrinsically Conductive and Cu-Functionalized Polymer-Composite Membranes as Gas Diffusion Electrodes for CO_2 Electroreduction

Ignacio Sanjuán[a,d], Vaibhav Kumbhar[a,d], Oleg Prymak[c,d], Mathias Ulbricht[b,d,e], Corina Andronescu[a,d,e], Lukas Fischer[b,d,e]*

[a]*Technische Chemie III, Universität Duisburg-Essen, Carl-Benz-Straße 199, 47057 Duisburg, Germany*

[b]*Lehrstuhl für Technische Chemie II, Universität Duisburg-Essen, Universitätsstr. 7, 45141 Essen, Germany*

[c]*Anorganische Chemie, Universität Duisburg-Essen, Universitätsstr. 5, 45141 Essen, Germany*

[d]*Center for Nanointegration Duisburg–Essen (CENIDE) and NanoEnergieTechnikZentrum (NETZ), Universität Duisburg-Essen, Carl-Benz-Str. 199, 47057 Duisburg, Germany*

[e]*Center for Water and Environmental Research (ZWU), Universität Duisburg-Essen, Universitätsstr. 2, 45141 Essen, Germany*

**Corresponding author. Lukas.Fischer@uni-due.de*

S1. Materials

N-methyl-2-pyrrolidone (NMP) (99%), copper(II) chloride (99%), copper(II) acetate (98%), $NaBH_4$ (99%), H_2SO_4 (98%, HPLC quality), KOH (86.9%), graphene nanoplatelets, carbon nanofibers (100 nm × 20-200 µm) and carbon nanotubes (multi-walled, 6-13 nm × 2.5-20 µm) were purchased from Sigma-Aldrich. Carbon nanoparticles (Vulcan XC72) were obtained from the Fuel Cell Store, polyethersulfone (PES) Ultrason® 6020P from BASF and ethanol (99%) from VWR. KOH solutions were purified before use by a cation-exchange resin in the potassium form (Chelex 100, Sigma Aldrich). All aqueous solutions were prepared with ultrapure water of 18.2 MΩcm (Millipore purification system).

S2. Membrane fabrication and conductivity measurement

For the optimization of the fabrication process, a base casting solution of 15 wt% PES in NMP was used. The respective fillers were added to the PES solution (wt% of fillers refers to the weight of the base solution), followed by either stirring for 24 h or high-shear mixing for 2 h. For the latter, the casting dispersion was placed in a 50 mL plastic centrifuge tube and was high-shear mixed at 3150 rpm using a neoLab 7-2020 vortex mixer. Afterwards, the casting dispersions were degassed for 15 min at 50 mbar, followed by film casting onto a glass plate with a film thickness of 200 µm. Then, the cast film was immediately immersed in a water coagulation bath for 10 min and the final membrane was stored in fresh DI water. For determination of the electrical conductivity, a self-made 2-point contact probe was used (Figure S1).

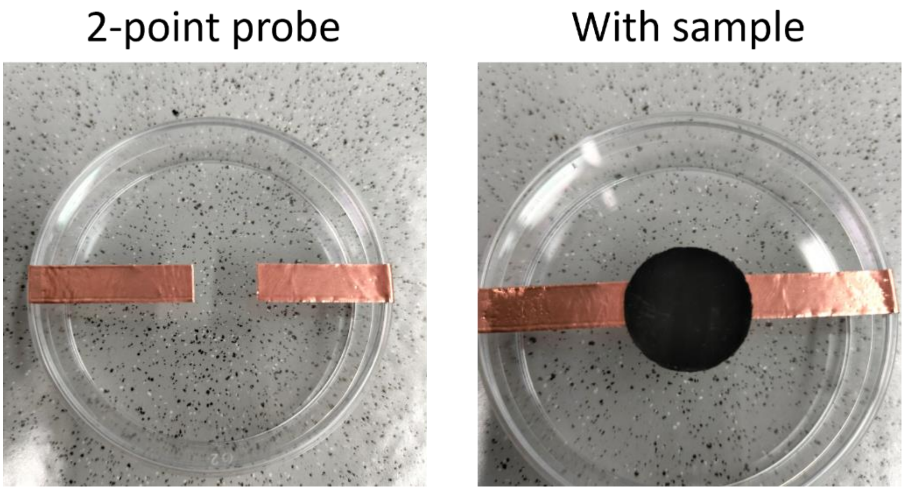

Figure S1: Self-made 2-point conductivity probe.

For the 2-point probe, we placed two strips of adhesive copper tape on top of a plastic petri dish. The two strips were 1 cm apart and had a width of 0.6 cm. The dry membrane sample was placed between the copper strips and a small plastic cube was placed on top of the membrane sample (~5 g/cm^2). Then, the resistance was determined at the copper strips using a digital multimeter M-4630 from Metex. The resistivity was calculated as follows:

$$\rho = \frac{R * T * 0.6\, cm}{1\, cm} \tag{1}$$

*ρ: Resistivity (Ohm*cm), R: Measured resistance (Ohm), T: Membrane thickness.*

The specific conductivity was calculated as 1/*ρ*. As control material, we used Panex PX30 carbon fiber fabric from Zoltek. With our setup, we determined 0.038 Ohm*cm for Panex PX30, compared to 0.022 Ohm*cm found in literature.[1]

All fabricated membranes are listed in Table S1 with their respective relative conductivity.

Table S1: Filler and condition used for the membrane fabrication as well as the mechanical integrity and relative conductivity of the resulting membranes. In red the final condition is marked.

**Filler**	**Condition**	**Mechanical Integrity**	**Relative conductivity**
4 wt% Carbon nanoparticles	Stirring	High	1
8 wt% Carbon nanoparticles	Stirring	Low	450
10 wt% Graphene	Stirring	High	50
2 wt% Carbon nanotubes	Stirring	Very low	700
4 wt% Carbon nanofibers	Stirring	Very low	400
8 wt% Carbon nanoparticles	High-shear mixing	High	500
8 wt% Carbon nanoparticles	+ 1 wt% Fumion	High	300
8 wt% Carbon nanoparticles	+ 1 wt% PVP	High	900
8 wt% Carbon nanoparticles	+ 1 wt% SDS	High	2700
10 wt% Carbon nanoparticles	+ 1 wt% SDS	Low	1200
8 wt% Carbon nanoparticles + 0.5 wt% Carbon nanofibers	+ 1 wt% SDS	Low	6400

The final base membranes were made with the condition shown in red in Table S1 and the casting dispersions were prepared as described above.

The anisotropic base membranes were fabricated via liquid non-solvent induced phase separation (NIPS). After casting, the cast film was immediately immersed in a water coagulation bath for 10 min and the final membrane was stored in fresh DI water afterwards. Overall, two membranes were made with film casting thicknesses of 150 µm and 120 µm, yielding thicknesses after solidification of ~140 µm (used for *An_CuEt*) and ~115 µm (used for *An_CuAq*), respectively.

The isotropic base membrane was fabricated via non-solvent vapor induced phase separation (VIPS). After casting with a film thickness of 200 µm, the cast film was immediately placed in a water vapor saturated chamber for 5 min, followed by immersion in a water coagulation bath for 10 min. Then, the final membrane was stored in fresh DI water. The thickness of the final solidified membrane was ~150 µm (used for *Iso_CuAq)*.

For these anisotropic and isotropic base membranes, we determined resistivities (Figure S1 and Equation 1) between ~5-7 Ohm*cm, corresponding to specific conductivities between ~0.14-0.2 S/cm.

S3. Copper coating procedure

The base membranes as described in Section S2 were used for the copper coating.

To determine the copper loading, membrane samples were dried after a completed coating step and the dry weight after coating was compared to the dry weight before coating. To determine the conductivity, membrane samples were dried after a completed coating step and the conductivity was measured as described in Section S2.

An_CuAq and *Iso_CuAq* were prepared by an aqueous iterative copper coating. An ethanol-wetted base membrane sample was incubated in 0.05 M $CuCl_2$ in water for 2 min. Afterwards, the sample was taken out of the solution and was stripped from excess solution on the surface. Then, the sample was placed in 0.02 M $NaBH_4$ in water for 2 min, followed by washing in ethanol for 2 min. After washing, the sample was used for the next coating step.

An_CuEt was prepared by an ethanol-based iterative copper coating. A water-wetted base membrane sample was incubated in 0.05 M $Cu(OAc)_2$ in ethanol for 2 min. Afterwards, the sample was stripped from excess solution on the surface and dried for 5 min at 50°C. Then, the sample was placed in 0.02 M $NaBH_4$ in ethanol for 2 min, followed by washing in water for 2 min. After washing, the sample was used for the next coating step.

Table S2: Cu loading and electrical conductivity of the final Cu-functionalized GDEs (average and standard deviation from three different 14 mm diameter samples).

	Cu loading [mg/cm^2]	Electrical conductivity [S/cm]
An_CuAq	1.60 ± 0.11	18.2 ± 2.5
Iso_CuAq	1.76 ± 0.17	17.7 ± 3.5
An_CuEt	0.51 ± 0.04	14.1 ± 1.2

S4. Copper coating of bare PES membrane

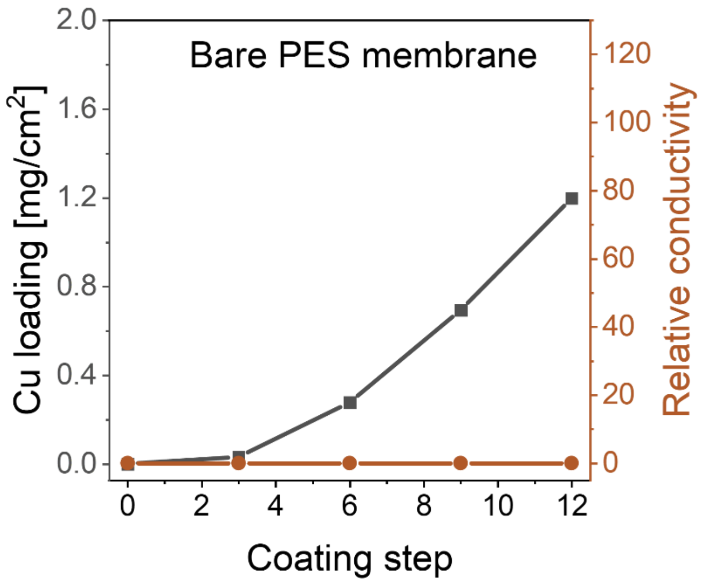

Figure S2: Iterative copper coating of a bare PES membrane without carbon filler. Coating was conducted in aqueous $CuCl_2$ and aqueous $NaBH_4$ as described in Section S3.

S5. Material characterization

For SEM and EDX analysis, dry samples of the base membranes and the Cu-functionalized GDEs were used. To access the cross section, samples were frozen in liquid nitrogen and then broken. Before analysis, samples were sputter coated with a PdAu alloy. SEM and EDX analyses were conducted on an Apreo S LoVac system from Thermo Fisher Scientific. The SEM images were recorded at a pressure of 10^{-3} Pa, an accelerating voltage of 5 kV and a current of 13 pA. For EDX mapping, an accelerating voltage of 30 kV was used.

For XPS, dry samples of the base membranes and the Cu-functionalized GDEs were used. XPS analysis was conducted on the top surface of the base membrane and the Cu-functionalized GDEs (contact to water precipitation bath during fabrication) on a PHI VersaProbe II with an Al Kα X-ray source resulting in a photon energy of 1486.6 eV.

For the XRD analysis, dry samples of the base membranes and the Cu-functionalized GDEs (24 mm diameter) were fixated on a flat silicon single crystal sample holder and measured with a Panalytical Empyrean instrument in reflection mode using Cu Kα radiation (λ = 1.54 Å; 40 kV, 40 mA). The samples were investigated from 5 to 90° 2Θ with a step size of 0.006° and a scan speed of 0.024 °/s resulting in a total measurement time of 60 min. Qualitative phase analysis was performed with Diffrac.Suite EVA V1.2 from Bruker with the pattern of Cu (#04-0836) from the ICDD database. Quantitative Rietveld refinement was performed with the Bruker software TOPAS 5.0 to calculate the lattice parameter a, the unit cell volume V and the average crystallite size CS from diffraction peak broadening (Table S3). For this, the instrumental peak correction as

determined with the LaB6 standard reference material (SRM 660b of NIST) was considered.

Table S3: Lattice parameter and crystallite size (CS) calculated from the refined XRD patterns.

	An_CuAq	*Iso_CuAq*	*An_CuEt*
a [Å]	3.6157	3.6157	3.6160
V [Å^3]	47.267	47.271	47.290
CS [nm]	53	54	29

S6. Capillary flow porometry

For the porometry analysis, dry samples of the base membranes and the Cu-functionalized GDEs were first wetted with Galwick (short chain length polymerized hexafluoropropene). The wetted samples were then placed in a capillary flow porometer (CFP-34RTG8A-X-6-LA) from PMI. Then, the liquid displacement pressure profile was determined by first measuring the volumetric gas flow at step-wise increasing nitrogen gas pressures for the wetted sample, followed by measuring the gas flow at increasing nitrogen gas pressures for the fully dewetted sample. For each nitrogen gas pressure point that led to liquid displacement, the diameter of the corresponding through-pores can be calculated with Washburn's equation as follows:

$$d = \frac{4\gamma}{P} \cdot \cos\theta \qquad (2)$$

d: Through-pore diameter, γ: Surface tension of the liquid, P: Transmembrane gas pressure difference, θ: Contact angle of liquid inside pores.

When using Galwick (perfluorinated liquid), a contact angle of 0° can be assumed and the through-pore diameter (Figure S3a) can be directly calculated (equation 2) from the displacement pressure (Figure S3b).

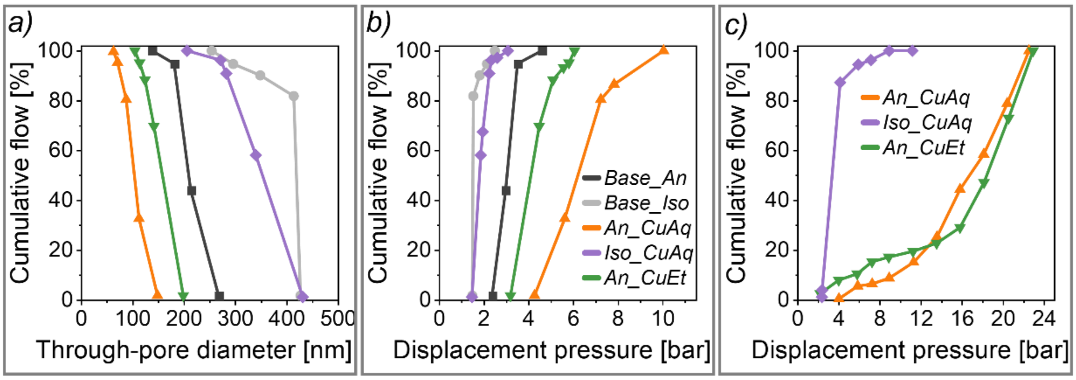

Figure S3: Porometry analysis of the base membranes and the Cu-functionalized GDEs. a) Calculated through-pore diameter using Galwick as wetting liquid. b) Determined displacement pressure using Galwick as wetting liquid. c) Determined displacement pressure using water as wetting liquid.

When using water as wetting liquid, the displacement pressure is influenced by both the through-pore diameter and the water contact angle in each through-pore (Figure S3c). Since the through-pore diameter is known from the analysis with Galwick, the effective contact angle of water inside the through-pores can be calculated from the data in Figure S3 using equation 2 (Table S4).

Table S4: Calculation of the effective water contact angle (θ) inside the through-pores of the Cu-functionalized GDEs.

		Diameter Galwick [nm]	Pressure water [bar]	*4γ/d* [N/m²]	*4γ/d* [bar]	*P*d/4γ* [bar/bar]	θ inside through-pores [°]
End of liquid displacement	*An_CuAq*	63	22.6	4598425	46.0	0.49	60
	An_CuEt	105	23.0	2775665	27.8	0.83	34
	Iso_CuAq	206	11.2	1411309	14.1	0.80	37
Start of liquid displacement	*An_CuAq*	149	4.1	1955793	19.6	0.21	78
	An_CuEt	199	2.2	1462925	14.6	0.15	81
	Iso_CuAq	432	2.4	674676	6.7	0.36	69

S7. Surface water contact angle

For the surface water contact angle and the water droplet uptake, dry samples of the base membranes and the Cu-functionalized GDEs were used. Analysis was conducted on an OCA15 system from Dataphysics. 3 µL DI water was deposited on the sample surface and the contact angle as well as the water droplet volume were dynamically recorded each 2 seconds for 40 seconds by the Dataphysics software. In Figure S4 the relative decrease of both the water droplet volume and the surface water contact angle (CA) over time is shown.

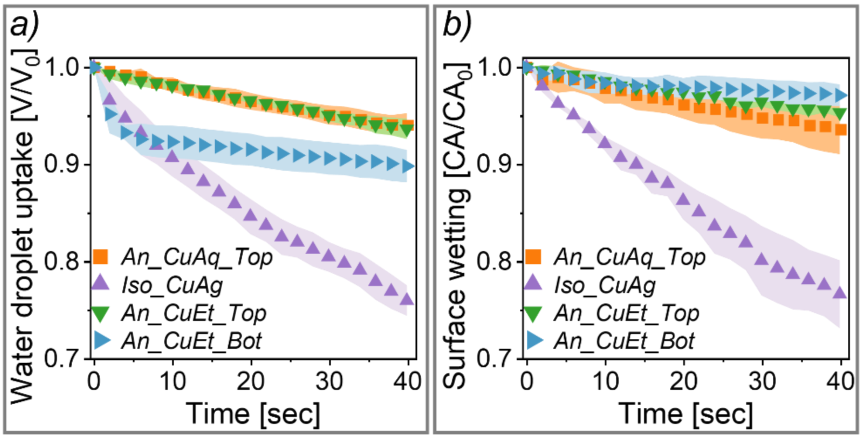

Figure S4: Relative decrease of the water droplet volume (a) and the surface water contact angle (b) over time for the Cu-functionalized GDEs.

S8. Electrochemical CO_2 reduction reaction

Electrochemical set-up. The electrochemical measurements were performed with a VSP-300 potentiostat (BIOLOGIC) in a three-compartment PEEK flow cell made in-house (Figure S3). An anion exchange membrane (AEM, FAB-PK-75, Fumatech) separated the cathode and anode compartments. The AEM was activated in 1 M KOH

for at least 24 h before use. The working electrode (GDE membrane) was placed between the gas and catholyte compartment (geometric area = 0.95 cm^2). The reference electrode (RE) was a leak-less Ag/AgCl/3.4 M KCl (Innovative Instruments). The counter electrode (CE) was a Ni foam (purity 99.5%, Goodfellow). 12 mL of 1 M KOH (pH 13.9) were used as catholyte and anolyte which were recirculated at 4 mL min^{-1} by a peristaltic pump (MINIPULS3, Gilson). The gas and cathode compartments were air-tight and connected to a gas chromatograph (GC).

CO_2RR experiments. CO_2RR experiments were performed galvanostatically. We applied increasing negative current densities in series (from -23 to -292 mA/cm^2), for 420 seconds each. 0.95 cm^2 were used as geometric area to calculate the current densities. Galvanostatic electrochemical impedance spectroscopy (GEIS) was carried out after each chronopotentiometry at the same current density, with frequencies between 100 kHz and 10 Hz, and a modulation current of 10% of the base current. During the experiment, CO_2 (Air Liquid, 99.9995%) flowed through the gas compartment with 20 mL min^{-1} and a N_2 flow (Alphagaz 99.99%) of 20 mL min^{-1} was continuously bubbling into the catholyte reservoir. The flow rate of the gases was controlled by mass flow controllers (EL-FLOW Select F-201CV, Bronkhorst®). Both gas streams (N_2 and CO_2) were mixed after the cell. A mass flow meter (35302L, Analyt-MTC) was placed between the cell and the GC to ensure the absence of leakages and to control the rate at which the reaction products reached the GC. The flow meter was calibrated for N_2 by default, so that when the N_2/CO_2 stream was measured, the actual flow rate was 87% of the displayed value (internal calibration).

All potentials were converted to the reversible hydrogen electrode (RHE) scale using the equation 2:

$$E_{iR}\ (RHE) = E_{Ag/AgCl} + E^{o}_{Ag/AgCl} + 0.059 \cdot pH - iR_u \qquad (3)$$

where E is the potential in the RHE scale (in V); $E_{Ag/AgCl}$ is the measured potential (in V versus Ag|AgCl|3.4 M KCl), and $E^{o}_{Ag/AgCl}$ is the standard potential of the Ag|AgCl|3.4 M KCl RE (0.21 V). E_{iR} values are 100% manually corrected for ohmic drop using the uncompensated resistance (R_u, Ω), which was calculated through GEIS in the high-frequency range of the Nyquist plots. The pH of 1 M KOH was calculated considering the OH^- concentration (C_{OH^-} in mol L^{-1}) and their activity coefficient in water (γ = 0.766 [57,58]):

$$pH = 14 + \log(C_{OH^-}) + \log\gamma \qquad (4)$$

Gas products analysis. Gas products were analyzed by gas chromatography. We used an SRI 8610C MG1 (SRI Instruments) GC equipped with a packed 3-meter HaySep D column. The GC allowed for automatic injections using an autosampler function and a 1 mL sample loop. The carrier gas was pure N_2 and the column temperature was 91°C. The detectors were a thermal conductivity detector (TCD) and a flame ionization detector (FID) with a methanizer device. The TCD quantified H_2 whereas the FID-methanizer analyzed CO, CH_4, C_2H_4, and C_2H_6. The GC was calibrated with a commercial gas mixture. During CO_2RR experiments, gas products were analyzed every 7.5 min. Every GC measurement lasted 6 min in total, with an additional stand-by time of 1.5 min before the injection to ensure that the gases reached the GC sample loop. The faradaic efficiency (FE) for a gas product "*i*" was calculated by equation 5:

$$FE_i = \frac{C_i^{gas} \cdot f_{gas} \cdot z_i \cdot F \cdot 10^{-9}}{I_t \cdot V_m \cdot 60} \cdot 100 \quad (5)$$

where I_t is the current (A); C_i^{gas} is the gas "*i*" concentration (ppm), f_{gas} is the flow rate (mL min^{-1}), z_i is the number of electrons transferred per each mol of "*i*"; *F* is the Faraday constant (96485 C per mol of electrons) and V_m is the molar volume of a gas at 1 bar and 25ºC (24.4 L mol^{-1}).

Liquid products analysis. Liquid products were quantified by high-performance liquid chromatography (HPLC). The HPLC was an AZURA HPLC system (Knauer) with a refractive index detector (RID 2.1L, Knauer) and a diode array detector (UV/VIS, DAD 2.1L, Knauer). The product separation was made by an anion exclusion column (Eurokat H, Knauer) and the respective precolumn, using a 5 mM H_2SO_4 solution as eluent. The eluent flow rate was 0.6 mL min^{-1} and the temperature was 70°C. For the analysis, 440 µL of electrolyte were taken directly from the cell at the end of every current density. Samples were acidified using a 2.5 M H_2SO_4 solution in a 4:1 volumetric ratio before their injection into the HPLC. The samples were maintained in an ice bath to avoid evaporation of volatile compounds due to the exothermic reaction occurring upon the addition of H_2SO_4. For each HPLC measurement, a 10 µL sample was injected using an auto-sampler. Products were assigned by calibration with commercially available reference compounds. The FE of a liquid product "*k*" was calculated by equation 6:

$$FE_k = \frac{C_k^{HPLC} \cdot V \cdot z_k \cdot F \cdot 10^{-6}}{I_t \cdot t} \cdot 100 \quad (6)$$

where C_k^{HPLC} is the concentration of "*k*" (mmol L^{-1}), *V* is the catholyte volume (mL) and *t* is the electrolysis time (seconds).

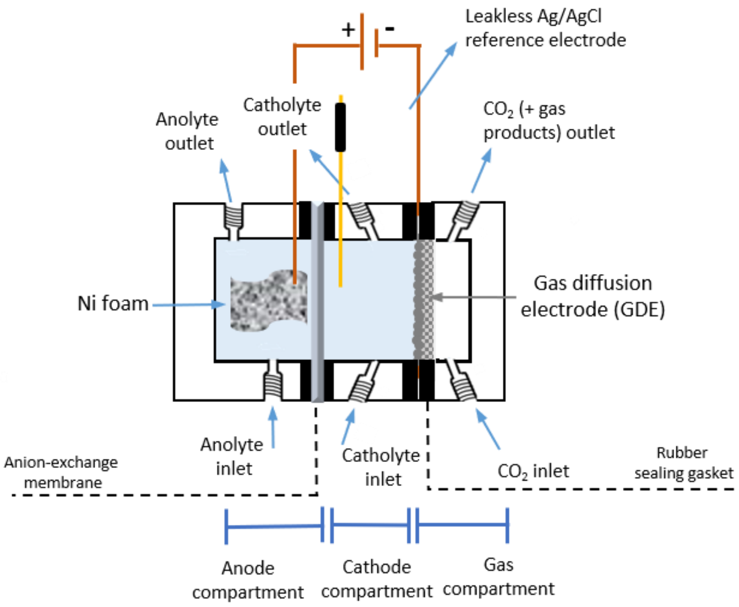

Figure S5: Scheme of the three-compartment electrochemical flow cell made of PEEK used in the CO_2RR experiments.

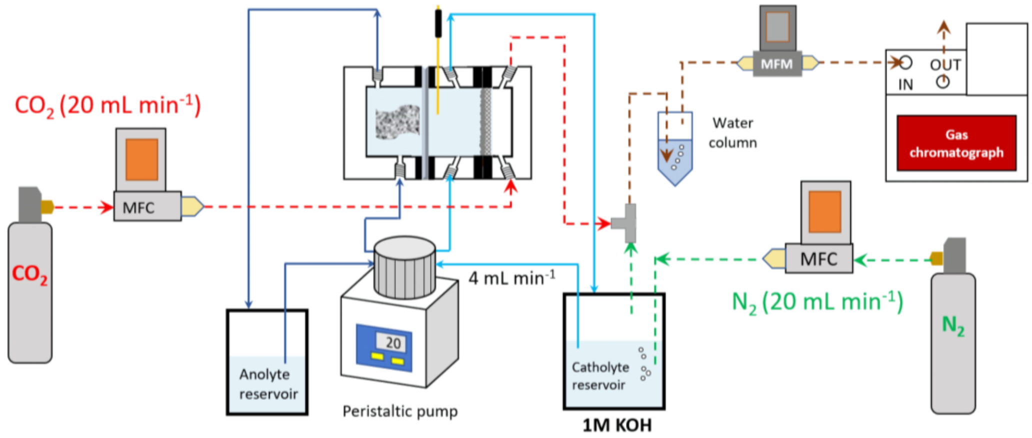

Figure S6: Scheme of the experimental set-up for CO_2RR experiments.

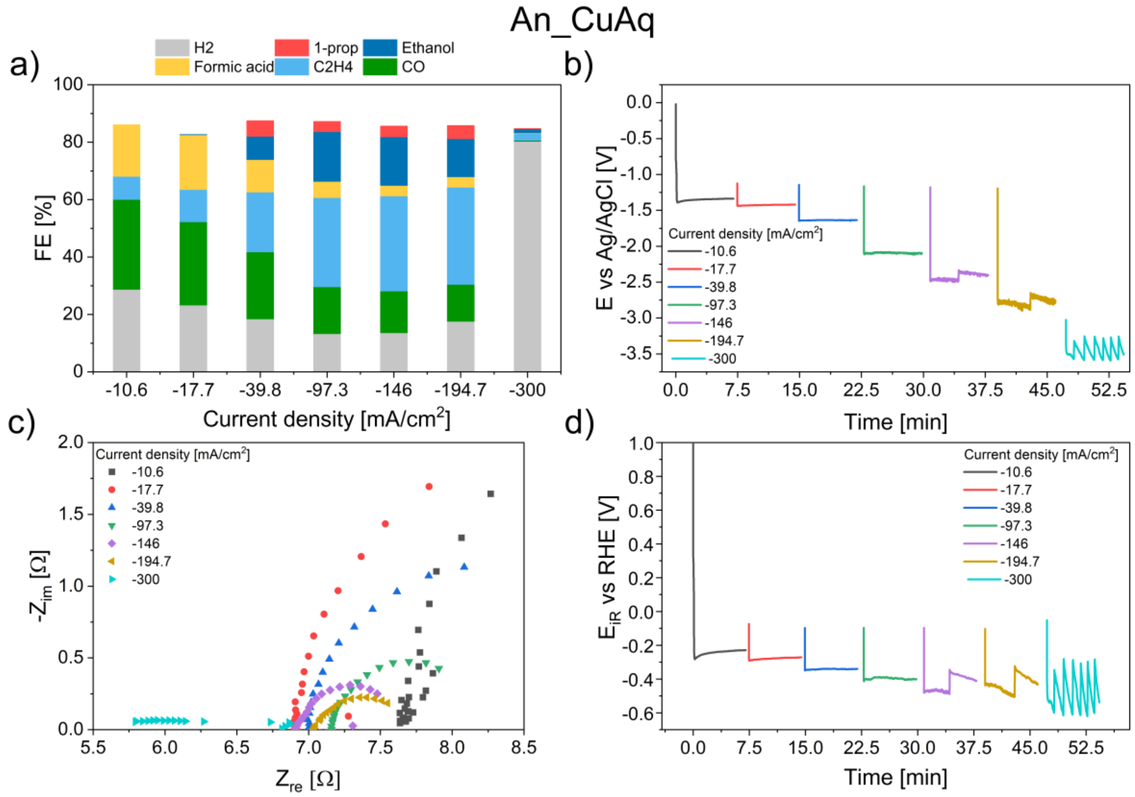

Figure S7: CO_2RR experiment for An_CuAq with the top side in contact to 1 M KOH. a) Faradaic efficiency for various products at different current densities. b) Evolution over time of the potentials versus Ag/AgCl at each current density (7.5 min each). c) Nyquist plots obtained by GEIS at different current densities. d) Evolution of the IR-corrected potentials versus RHE during the 7.5 min application of each current density.

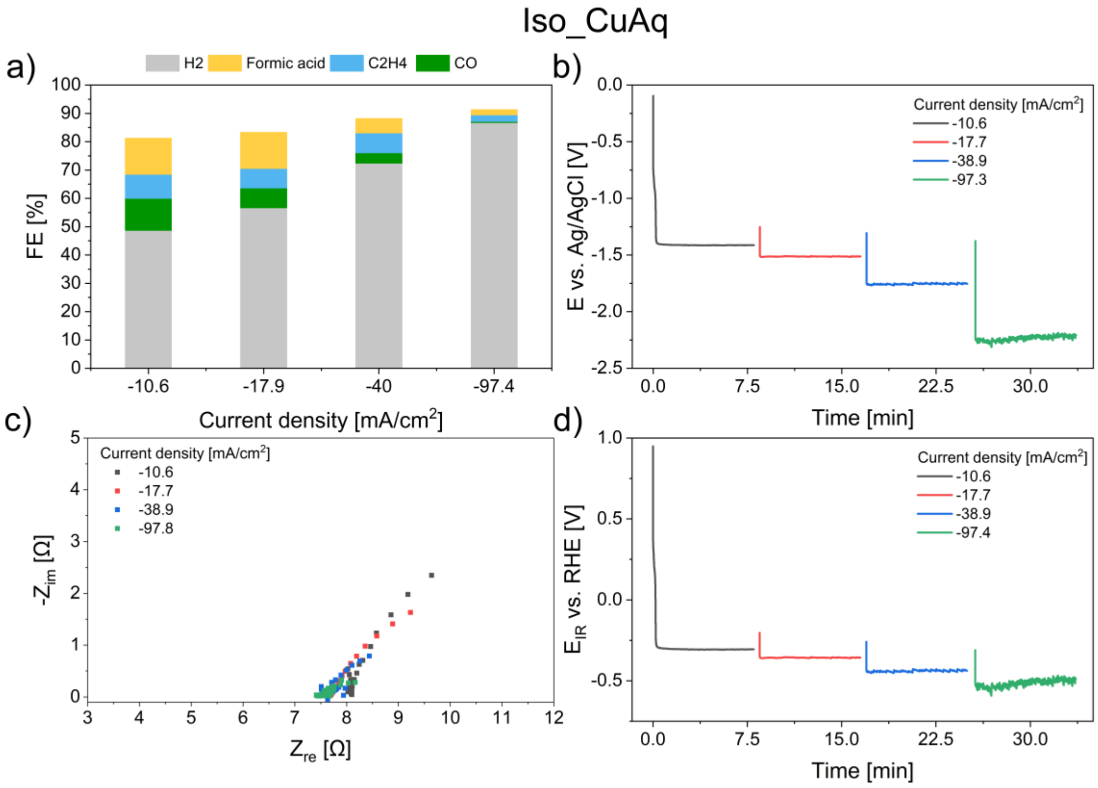

Figure S8: CO_2RR experiment for Iso_CuAq with the top side in contact to 1 M KOH. a) Faradaic efficiency for various products at different current densities. b) Evolution over time of the potentials versus Ag/AgCl at each current density (7.5 min each). c) Nyquist plots obtained by GEIS at different current densities. d) Evolution of the IR-corrected potentials versus RHE during the 7.5 min application of each current density.

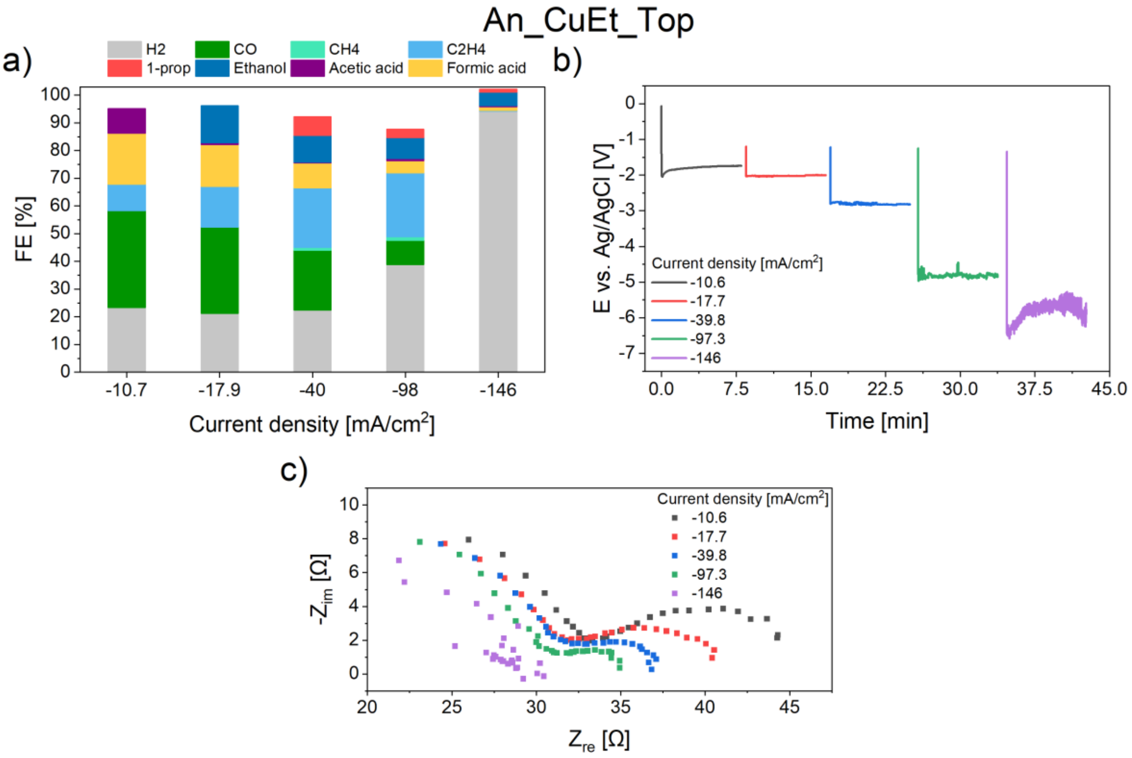

Figure S9: CO_2RR experiment for An_CuEt_Top with the top side in contact to 1 M KOH. a) Faradaic efficiency for various products at different current densities. b) Evolution over time of the potentials versus Ag/AgCl at each current density (7.5 min each). c) Nyquist plots obtained by GEIS at different current densities. The Nyquist plot profiles registered for this electrode were anomalous, with a hemi-circle observed at high frequencies. Due to the lack of quality assurance, the potentials for this GDE were not IR-corrected.

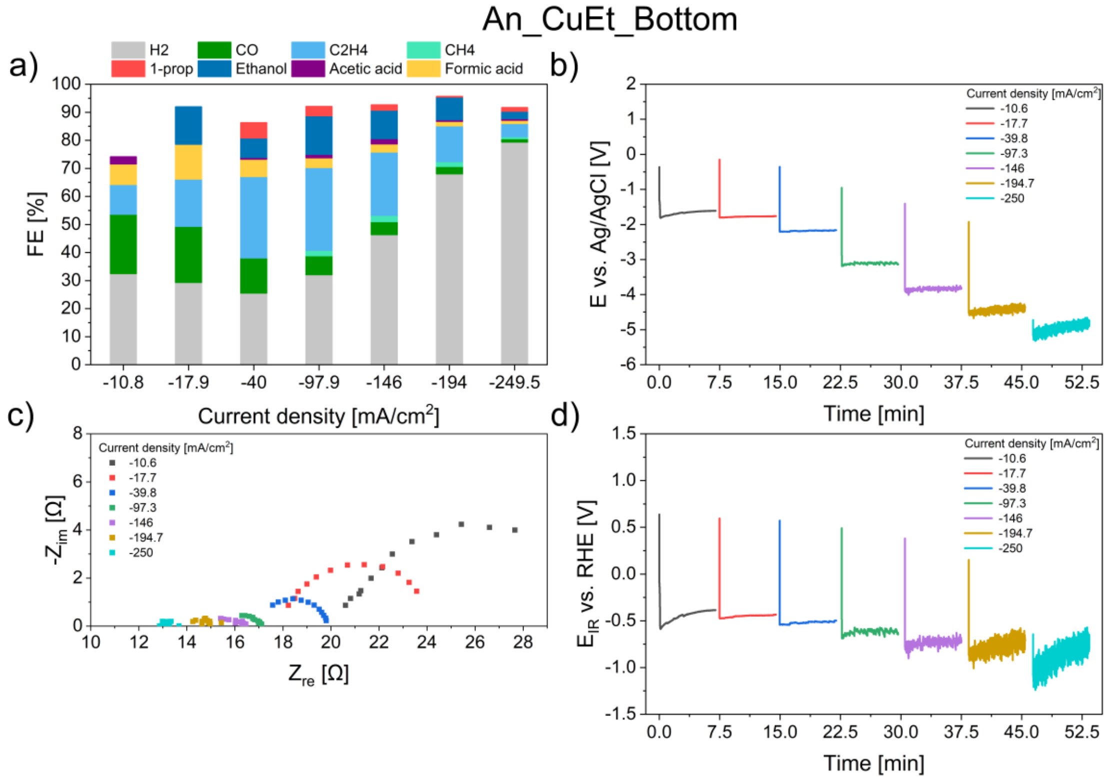

Figure S10: CO_2RR experiment for An_CuEt_Bot with the bottom side in contact to 1 M KOH. a) Faradaic efficiency for various products at different current densities. b) Evolution over time of the potentials versus Ag/AgCl at each current density (7.5 min each). c) Nyquist plots obtained by GEIS at different current densities. d) Evolution of the IR-corrected potentials versus RHE during the 7.5 min application of each current density.

References

(1) Snyder, J. F.; Wong, E. L.; Hubbard, C. W. Evaluation of Commercially Available Carbon Fibers, Fabrics, and Papers for Potential Use in Multifunctional Energy Storage Applications. *J. Electrochem. Soc.* **2009**, *156* (3), A215. https://doi.org/10.1149/1.3065070.
